# A microdeletion event at 19q13.43 in IDH-mutant astrocytomas is strongly correlated with MYC overexpression

**DOI:** 10.1186/s40478-024-01811-1

**Published:** 2024-06-14

**Authors:** Ege Ülgen, Umut Gerlevik, Sıla Gerlevik, Yavuz Oktay, Osman Uğur Sezerman, Şevin Turcan, Koray Ozduman

**Affiliations:** 1 Department of Biostatistics and Medical Informatics, School of Medicine, Acibadem University, Istanbul, Turkey; 2 Department of Neurosurgery, School of Medicine, Acibadem University, 34752 Istanbul, Turkey; 3 Department of Biochemistry, University of Oxford, Oxford, UK; 4 Faculty of Medicine, Ludwig Maximilian University of Munich, Munich, Germany; 5 Faculty of Life Sciences and Medicine, Comprehensive Cancer Centre, School of Cancer and Pharmaceutical Sciences, King’s College London, London, UK; 6 Izmir Biomedicine and Genome Center, Izmir, Turkey; 7 Department of Medical Biology, Faculty of Medicine, Dokuz Eylül University, Izmir, Turkey; 8 Neurology Clinic and National Center for Tumor Diseases, Heidelberg University Hospital and Heidelberg University, Heidelberg, Germany

**Keywords:** Integrative analysis, Gliomagenesis, MYC dysregulation, Astrocytoma, 19q deletion

## Abstract

MYC dysregulation is pivotal in the onset and progression of IDH-mutant gliomas, mostly driven by copy-number alterations, regulatory element alterations, or epigenetic changes. Our pilot analysis uncovered instances of relative MYC overexpression without alterations in the proximal MYC network (PMN), prompting a deeper investigation into potential novel oncogenic mechanisms. Analysing comprehensive genomics profiles of 236 “IDH-mutant 1p/19q non-co-deleted” lower-grade gliomas from The Cancer Genome Atlas, we identified somatic genomic alterations within the PMN. In tumours without PMN-alterations but with MYC-overexpression, genes correlated with MYC-overexpression were identified. Our analyses yielded that 86/236 of astrocytomas exhibited no PMN-alterations, a subset of 21/86 displaying relative MYC overexpression. Within this subset, we discovered 42 genes inversely correlated with relative MYC expression, all on 19q. Further analysis pinpointed a minimal common region at 19q13.43, encompassing 15 genes. The inverse correlations of these 15 genes with relative MYC overexpression were re-confirmed using independent scRNAseq data. Further, the micro-deleted astrocytoma subset displayed significantly higher genomic instability compared to WT cases, but lower instability compared to PMN-hit cases. This newly identified 19q micro-deletion represents a potential novel mechanism underlying MYC dysregulation in astrocytomas. Given the prominence of 19q loss in IDH-mutant gliomas, our findings bear significant implications for understanding gliomagenesis.

**Supplementary Information:**

The online version contains supplementary material available at 10.1186/s40478-024-01811-1.

## Introduction

MYC dysregulation plays a central role in gliomagenesis. In astrocytomas, a subset of gliomas characterized by mutations in the isocitrate dehydrogenase (IDH) gene, relative MYC overexpression is one of the core driver events [1]. In these tumours, several single nucleotide polymorphisms (SNP) lead to overexpression of MYC and these are associated with the highest odds ratios reported in any cancer type [2–4]. Copy number gains of MYC are commonly observed in IDH-mutant gliomas [5]. Malignant degeneration is strongly correlated with increased MYC signalling [6]. In addition to MYC, alterations of the MYC paralogs, i.e. MYC-N or MYC-L, are commonly observed in childhood gliomas. MYC influences several essential processes, including gene expression programs, cell cycle, DNA repair, genomic stability, and cell metabolism, among several others, and is a key mediator of central nervous system stem cells [7–12]. There is experimental evidence that MYC plays a role in maintaining glioma cancer stem cells and regulating intercellular competition during early stages of gliomagenesis [13, 14].

The mechanisms of action and regulation of MYC are complex and not completely understood. Nevertheless, MYC, its two paralogs and several other direct acting regulatory genes are grouped together as the proximal MYC network (PMN), where several alterations can substitute for each other. Furthermore, the alterations that drive overexpression of MYC are also numerous, including simple copy number gains, germline alterations in regulatory elements, and epigenetic silencing. However, the mechanisms driving MYC overexpression have not been fully elucidated, and there remains a large proportion of astrocytomas that appear to lack any oncogenic alterations in PMN genes. With this motivation, using the publicly available The Cancer Genome Atlas (TCGA) lower-grade glioma cohort, we performed a systematic and integrative analysis of PMN genes in astrocytomas and identified alternative oncogenic events.

## Material and methods

### Overview of analyses

In this study, we conducted an analysis of publicly available data from The Cancer Genome Atlas (TCGA) to investigate the genomic changes within the proximal MYC network (PMN) in low-grade gliomas harbouring IDH mutations and lacking co-deletion of 1p/19q. Furthermore, we examined gliomas without any PMN alterations but presenting an elevated level of MYC transcription, aiming to unveil a novel mechanism responsible for relative MYC overexpression in these tumours. We identified a set of genes with somatic copy-number loss, all located in a narrow locus on the chromosomal arm 19q. Additionally, employing single-cell RNA sequencing data from distinct cases, we investigated the association between MYC expression, and the copy-number estimate of this locus in astrocytoma cells with and without copy-number loss events in the minimal common region (MCR).

### Data acquisition

The open-access data on lower-grade glioma (WHO grade II/III) from The Cancer Genome Atlas (TCGA) for the corresponding analyses were retrieved from GDC Data Portal [15]. This data was accessed using the R package TCGA biolinks (on 10.12.2023) [16, 17]. The dataset encompassed various types of information, including: (1) clinical data, (2) annotated simple nucleotide variation data from whole exome sequencing, (3) gene expression quantification derived from transcriptome sequencing (RNA-sequencing), and (4) copy number variation data from Affymetrix SNP 6.0 array (masked copy-number segments). Furthermore, additional curated survival data, including overall survival and progression-free interval, were obtained from the TCGA Clinical Data Resource [18].

PMN genes were collected from the pan-cancer study of PMN by Schaub et al. [5]. The PMN consists of “E-box transcription activation”-related genes (MYC, MYCN, MYCL, MLXIPL and MLXIP), “E-box transcription inhibition”-associated genes (FBXW7, MGA, MNT, MXD4, MXD3, MXI1 and MXD1), and “interacting/dimerization”-related genes (MAX and MLX) [5, 19]. While we attempt to provide a brief description of the roles of each PMN gene here, we encourage the reader to refer to the study by Schaub et al. for a more in-depth discussion. As explained by Schaub et al., all PMN proteins have related basic helix-loop-helix zipper (bHLHZ) domains and can be considered members of the “MYC bHLHZ” superfamily. These different components of the network are connected through dimerization with MAX, MLX, or both [5, 20–22]. Importantly, it has been shown that through dimerization with its partners (MLXIP and MLXIPL), the MAX-like protein MLX can either support or antagonize MYC function depending on cell context. In addition to the original set of genes in the PMN, we also included FBXW7, a gene demonstrated to be a critical regulator of MYC in the curated list of PMN genes [23, 24].

The single-cell data pertaining to IDH-mutant gliomas was sourced from the study conducted by Venteicher et al. (data accessible at NCBI GEO database, accession GSE89567) [25].

### Selection of samples for analysis

In our analyses, we selectively included only primary IDH-mutant and 1p/19 non-co-deleted gliomas, specifically referred to as “IDH-mutant astrocytoma”, which had complete data available (n = 236). To ensure the most current classification, we applied molecular grading criteria: If a tumour exhibited a homozygous deletion of CDKN2A or CDKN2B, it was assigned a molecular grade of “grade 4” [26]. Among the samples, there were 119 tumours classified as “grade 2”, 104 as “grade 3”, and 13 as “grade 4”. In line with previous studies, we observed a significant difference in overall survival between the various molecular grades (log-rank *p* < 0.001) [26, 27].

### Analyses of PMN alterations

In the initial phase of our study, we investigated the overall frequencies of alterations, encompassing somatic simple nucleotide variations (SNVs) and copy-number alterations (SCNAs), within the list of curated PMN genes. Using a threshold of 0.3, we defined SCNAs as events where the segment mean value (log ratio) is larger than 0.3 or less than 0.3, collectively including copy-number shallow/deep deletions or gains or amplifications. Furthermore, we analysed the alteration frequencies based on the molecular grade classification of the gliomas.

We assessed any association “PMN-hit” (defined as any somatic PMN alteration) with various other factors, including age, overall survival, progression-free interval, telomere maintenance mechanisms, and MGMT promoter methylation status.

Next, we assessed the associations of these alterations with various genomic instability metrics. These metrics, which we devised and described previously, were used to characterise various aspects of genomic instability, including SNV burden, Insertion/Deletion (InDel) burden, copy-number alteration frequency (wGII: weighted Genomic Instability Index), degree of aneuploidy (CAER: chromosomal arm event ratio), copy-number amplitude, and chromothripsis status [28].

Finally, we examined the MYC expression levels comparing by molecular grade, “PMN-hit” status, as well as the combination of the two.

### Monte Carlo simulation to assess the significance of the high number of PMN SCNAs

To determine whether the high number of SCNAs in PMN genes within our cohort of glioma samples was due to chance, we conducted a stratified permutation test, a subtype of Monte Carlo simulation. This approach allowed us to evaluate the extremity of the observed SCNA events against a null distribution generated by random sampling.

For each of 10,000 iterations, the following steps were performed:

1. Tumour sample stratification: We randomly selected a subset of tumour samples, ensuring that the number of samples matched the size of the original group that exhibited SCNA events in PMN genes.
2. Gene selection: Within each selected subset of tumour samples, we then randomly selected a set of genes. The number of genes selected matched the size of the original set of PMN genes observed to have SCNA events.
3. SCNA counting: For each random gene set within each subset of tumour samples, we counted the number of SCNA events.

By repeating this process for 10,000 distinct iterations, we generated a distribution of SCNA counts that could occur by random chance. We then compared the observed SCNA count in the PMN genes against this null distribution to calculate a *p* value, which quantified the likelihood that the observed high number of SCNAs in PMN genes could be attributed to random variation alone.

This permutation test enabled us to rigorously assess the statistical significance of our findings, controlling for potential biases arising from patient-specific variations and ensuring robust conclusions about the non-random nature of SCNAs in PMN genes.

### Analyses of “PMN-WT” cases with increased MYC expression

We next examined the “PMN-WT” samples (those without any somatic PMN alterations) to identify a set of genes that might potentially be regulators of MYC (a) whose expression levels are correlated with MYC expression level and (b) whose somatic alterations are associated with significantly increased MYC expression level.

For (a), the Pearson correlation coefficients and the associated significance values between the expression levels of MYC and all remaining genes were calculated. For any non-PMN gene, if the following condition was met: |r| > 0.2 and *p* < 0.05, the gene’s expression was accepted to be associated with MYC expression.

For (b), using the set of all somatically altered genes (in at least 10% of samples and at most 90% of samples), one-tailed Wilcoxon rank-sum tests were performed to determine if a given alteration was associated with higher MYC expression level (at *p* < 0.05). In determining both (a) and (b), we excluded commonly mutated astrocytoma genes (IDH1/2, TP53, ATRX, CDKN2A/B) and PMN genes.

We identified that the most frequent event in these potential MYC regulator genes was somatic copy-number loss events. Because these genes with copy-number loss were aggregated in a relatively small genomic region, we attempted to determine a minimal common region (MCR) with somatic copy-number loss within all of the altered samples (that are “PMN-WT” but have increased MYC expression) utilising the R package Genomic Ranges [29].

### Determination of the relationship between MCR status and MYC expression using single-cell RNA-sequencing data

We re-analysed the single-cell RNA-sequencing data of 5318 cells from 8 IDH-mutant astrocytomas by using the R package Seurat v5 [30] for data processing and clustering of cells, the python package SCSA for cell type annotation [31], and the R package CopyKat [32] for estimating the copy-number profiles of cells. For cell type annotation, the CellMarker 2.0 and CancerSEA databases were used [33, 34]. Using Seurat, we visualized the relationship between the copy number estimates of relevant intervals from CopyKat (those that overlap with the MCR identified in the previous step) and the RNA expression level of MYC, separately analysing these in the cells with and without MCR loss. Further, we estimated the effect of each region’s copy number estimate on MYC expression level (counts) in interaction with MCR status:

$$MYC_{{region_{i} }} = e^{{\beta_{0} + \beta_{1} CN_{{region_{i} }} \times I\left( {with\,MCR\,loss} \right) + \beta_{2} CN_{{region_{i} }} \times I\left( {without\,MCR\,loss} \right)}}$$

where “$MYC_{{region_{i} }}$” indicates the expression counts of MYC, “*region*_*i*_” is the CopyKat MCR region at index *i*, “CN” is the copy number estimate.

### Availability of analysis scripts

All scripts (along with basic documentation) used in the analyses for producing the findings in this study are publicly available on this GitHub repository: https://github.com/egeulgen/IDH-mut-astro-PMN-2024

## Results

### IDH-mutant astrocytoma cohort characteristics and the proximal MYC network

We analysed a dataset of 236 IDH-mutant astrocytoma samples, encompassing a spectrum of tumour grades with a distribution reflecting the prevalence of these grades in this tumour type: 119 Grade 2, 104 Grade 3, and 13 Grade 4. In this TCGA cohort of lower-grade gliomas, grade 4 tumours were called as such based on presence of CDKN2A/B deletion.

**Fig. 1:**
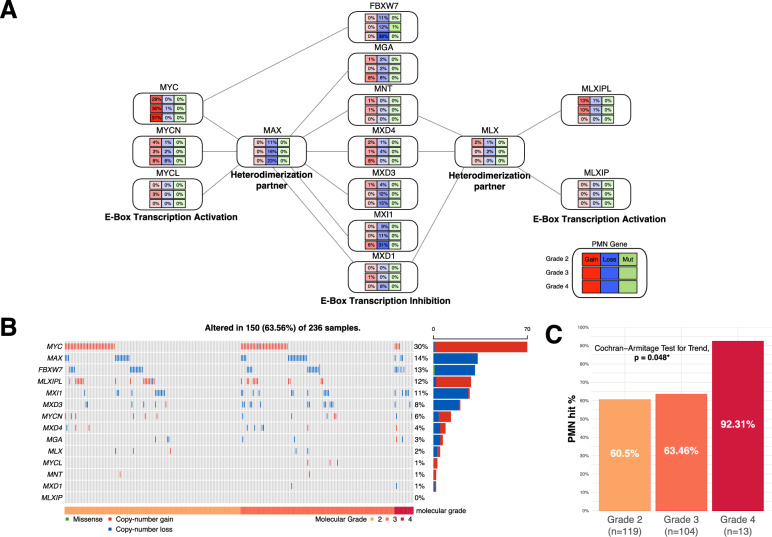
Distribution of the proximal MYC network (PMN) somatic alterations over the TCGA samples. **A** Percentages of samples with copy-number gains (leftmost), losses (middle), and mutations (rightmost) per gene in the PMN, each row corresponds to a molecular grade (in increasing grade). **B** Oncoprint for the PMN somatic alterations. **C** Bar plot displaying the percentages of samples with any somatic alteration in the PMN (“PMN-hit %”) by increasing grade, demonstrating increasing trend

When these tumours were analysed to investigate the prevalence of somatic alterations (mutations, gains, and losses), 150/236 (64%) of the samples were found to harbour at least one somatic alteration within the PMN (Fig. 1B). MYC, MYCN, MYCL and MLXIPL alterations primarily occurred as gains, aligning with their well-established roles as E-box activators (Fig. 1A, B). Contrarily, FBXW7, MXI1, MXD3, MXD4 and MGA were mainly observed as copy-number loss events, in line with their E-box inhibition functions. MAX also had copy-number loss in a significant number of samples, agreeing with its inhibitory function on MYC via heterodimerization. The gene with the most frequent copy-number gain was MYC which was observed in 30% of samples across all grades (Fig. 1B). MLXIPL also exhibited frequent copy-number gain, particularly in grades 2 and 3. MAX1, FBXW7, and MXI1 were identified as the most frequently genes with copy-number loss, and the rate of loss increase with grade. Mutations were only observed in FBXW7. As shown in Fig. 1A, B, the only 2 events observed in the MNT gene were copy-number gains. Similarly, there were very few alterations in MLX, MXD1 and MLXIP.

We observed 249 SCNA events in 13 PMN genes in 149 tumour samples, which is significantly higher than expected by chance (*p* = 0.0104).

### PMN hits increase parallel to tumour grade and are associated with lower progression-free interval and higher genomic instability

The frequency of any PMN-hit increased parallel to tumour grade (Fig. 1C, Cochran—Armitage Test, *p* = 0.048). PMN-hit status was not associated with a difference in overall survival (Supplementary Fig. 1B), but with a shorter progression-free survival (Supplementary Fig. 1B, C, log-rank *p* = 0.0048). There were no other significant associations between PMN-hit status and clinical variables such as age at diagnosis, telomere maintenance mechanism of the tumour, or MGMT promoter methylation status (Supplementary Fig. 1A, D, E, respectively).

In terms of genomic instability metrics, PMN-hit was found to be associated with increased SNV burden, copy-number alteration frequency, degree of aneuploidy and chromothripsis events but not with InDel burden and copy-number amplitude (Supplementary Fig. 2).

### MYC expression level is associated with PMN-hit status, independent of grade

**Fig. 2:**
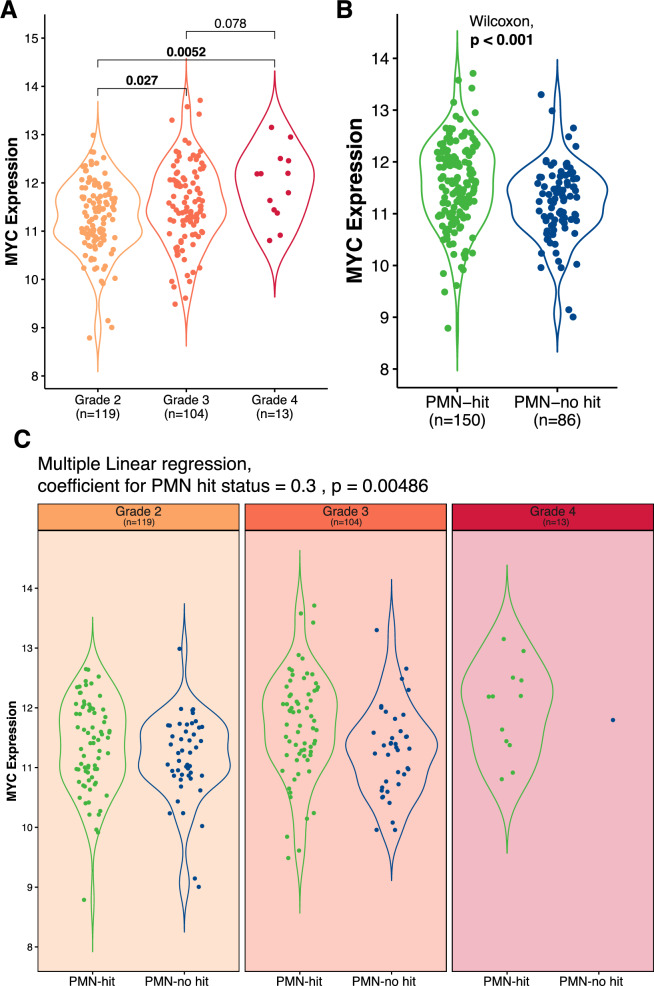
MYC expression level by **A** molecular grade, **B** PMN-hit, and **C** PMN-hit stratified by molecular grade

MYC expression also increased parallel to tumour grade (Fig. 2A). We also observed significantly higher MYC expression in tumours with PMN-hit compared to those with no PMN-hit (Fig. 2B). Fitting a linear regression model with PMN-hit status, adjusting for grade, revealed that PMN-hit was associated with increased MYC expression independent of grade (*p* = 0.00486).

### In cases without PMN-hit, recurrent loss events on chromosome 19q are associated with increased MYC expression

Within the subset of tumours without PMN-hits (PMN-WT cases, n = 86) we examined genes whose expression levels were correlated with MYC expression. We found 42 such genes whose alterations were inversely correlated with MYC expression. These alterations were somatic copy number loss events observed in 21/96 tumour samples. All of these genes with copy-number loss were located on chromosome 19q13.

**Fig. 3:**
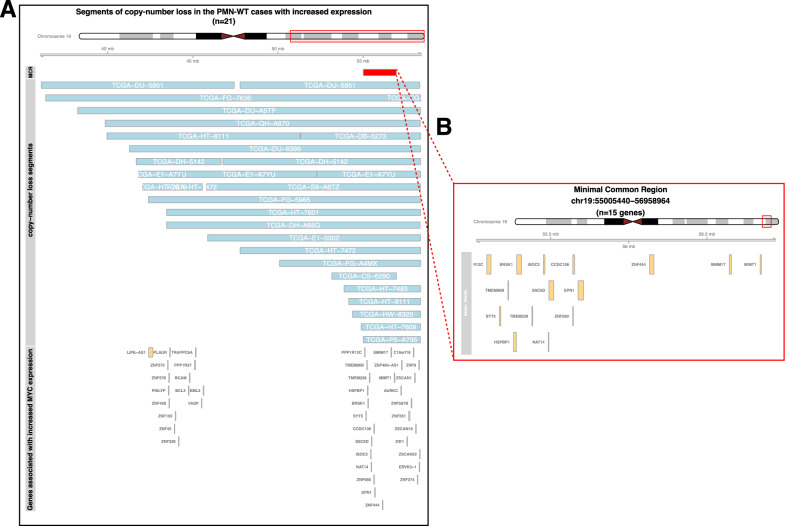
Minimal common region of copy-number loss of PMN-WT samples with increased MYC expression. **A** Minimal common region in the PMN-WT cases with increased MYC expression. **B** Genes contained within the MCR

Next, we determined the minimal common region (MCR) of copy-number loss (chr19: 55005440–56958964) in these samples, given their overlapping loss segments (Fig. 3A). The deleted MCR harbours 15 genes associated with increased MYC expression in these PMN-WT tumours (Fig. 3B). 13/15 of these genes displayed lower levels of expression in cases with MCR loss compared to cases with intact MCR (Supplementary Fig. 3).

Since chromosome 19q loss events are commonly observed in astrocytomas and because the MCR is in 19q, we examined the relationship between total 19q loss and MYC expression but found no significant association (Supplementary Fig. 4).

### Classification of IDH-mutant astrocytomas based on PMN-hit status and MCR loss status reveals 3 sub-classes with distinct characteristics

Using the PMN status and the newly identified MCR, we categorised all IDH-mutant astrocytomas into 3 sub-classes: (1) “PMN-hit” tumours (those with any somatic alterations in the PMN), (2) “MCR-loss” tumours (those without any somatic alterations in the PMN but with a copy-number loss event overlapping the MCR) and (3) “WT” tumours (those with neither events).

**Fig. 4:**
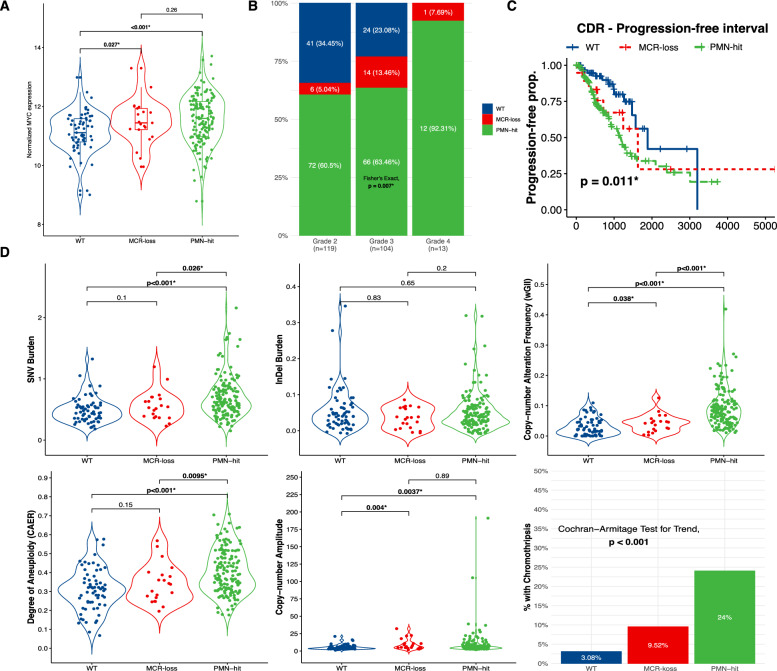
Comparison of subclasses. In terms of **A** MYC expression, **B** molecular grades, **C** progression-free interval, and **D** genomic stability metrics

We first analysed MYC expression by these sub-classes. PMN-hit cases displayed significantly higher MYC expression compared to both MCR-loss and WT cases (*p* < 0.001, Fig. 4A). MCR-loss tumours exhibited relatively increased MYC expression levels intermediate between PMN-hit and WT cases (Fig. 4A).

Next, we identified that this classification is additionally associated with tumour grade (Fisher’s exact, *p* = 0.007, Fig. 4B). As before, “PMN-hit” percentage increased with increasing grade, suggesting an association between PMN alterations and aggressive tumour behaviour. Notably, “MCR-loss” tumours also showed a trend towards higher grades compared to “WT” tumours.

We also observed a clinical association of this classification with progression-free interval (log-rank *p* = 0.011, Fig. 4C). Patients with “PMN-hit” tumours again demonstrated a significantly shorter progression-free interval compared to the rest, the “MCR-loss” subclass trended towards the “PMN-hit” subclass and “WT” subclass was observed to have the most favourable prognosis.

The different subclasses also differed in terms of genomic instability (Fig. 4D). “PMN-hit” tumours displayed higher genomic instability, as evidenced by increased SNV burden, InDel burden, copy-number amplitude, and chromothripsis frequency compared to MCR-loss and WT tumours. Interestingly, “MCR-loss” cases had intermediate levels of genomic instability compared to PMN-hit and WT groups, suggesting a potential association of the MCR loss with genomic integrity.

### Re-analysis of the data from a single cell study confirms the association between the minimal common region loss and MYC expression

To validate our finding in the TCGA IDH-mutant astrocytoma tumours that MYC expression is increased in tumours with a somatic copy number loss of the chromosome 19q MCR, we re-analysed single cell RNA-seq data from 8 IDH-mutant astrocytoma samples by Venteicher et al. [25]. When we estimated copy number log-ratios using CopyKat [32], we determined that copy number loss events overlapping the MCR were heterogeneous: they were not observed in all samples and were not observed in all cells per sample (Supplementary Fig. 5).

**Fig. 5:**
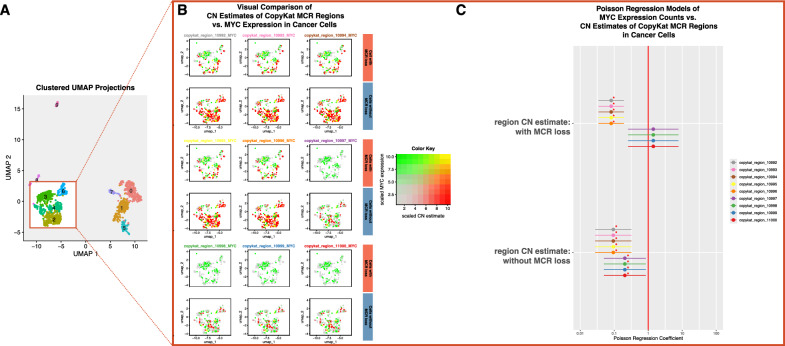
MYC expression increases upon minimal common region loss. **A** Clustered UMAP projections of the single cell data, the clusters in the squared region contain the cells of interest. **B** Association between MYC expression and copy number of the MCR regions in the cells with and without any loss. **C** Plot of estimated Poisson regression coefficients (dots) with 95% confidence intervals (lines) within the CopyKat MCR regions, estimating the effect of the region copy-number estimate on MYC expression for cells with and without any MCR loss (significant effects are indicated by red asterisks)

After clustering cells using Seurat [30], we annotated the cell types of each cluster using SCSA [31] (Fig. 5A). Clusters 0, 1, 5 and 7 were annotated as “Macrophages”, cluster 8 as “Endothelial cell”, cluster 9 as “Oligodendrocyte”, and clusters 2, 3, 4 and 6 as “Cancer stem cells”. To best investigate cancer-specific associations without any confounding effect from non-cancer cells, we used the “Cancer stem cells” clusters for further analyses.

Using the “Cancer stem cells”, we analysed the association between the copy-number estimates of the 9 MCR regions and the expression status of MYC separately visualising cells with and without MCR loss events (Fig. 5B). Specifically inspecting cancer cells with MCR loss, we observed that in the vast majority of cells, the copy-number estimate of the MCR regions were low whereas the expression level of MYC was increased.

We additionally examined the association between the aggregated expression of MCR genes and MYC expression within the same cancer cells. This also revealed that most cancer cells displayed low expression of MCR genes and elevated expression of MYC (Supplementary Fig. 6). This was further confirmed at single gene level, i.e. comparison of each MCR gene’s expression with MYC expression within the same cells (Supplementary Fig. 7).

## Discussion

The current analysis re-confirmed the presence of a PMN hits in roughly two-thirds of IDH-mutant astrocytomas. As previously demonstrated for gliomas in general, both the incidence of a PMN hit and the expression of MYC increased in parallel to the grade of the tumour in IDH-mutant astrocytomas. Presence of a PMN hit was not significantly associated with any clinical variable except for the progression free survival. Mutational metrics indicated that the “PMN-hit” group exhibited higher genomic instability, as indicated by significantly higher SNV burden, significantly higher CAN-frequency, significantly higher degree of genomic instability and significantly higher incidence of chromothripsis events (Supplementary Fig. 2). These findings may indicate to variations in oncogenic processes among IDH-mutant astrocytomas [28]

Further analysis into the “WT” group revealed a subset of 21 tumours which had 42 genes with their expression inversely correlated with MYC expression. All of these 42 genes had copy number losses and were localized to 19q. The minimal common region of loss was localized to 19q13.43 and had a somatic copy-number loss in all 21 tumours. This minimal common region contained 15 of the 42 genes associated with increased MYC expression. Further analyses using single-cell RNA sequencing from a separate dataset of seven IDH-mutant astrocytomas revealed that MYC expression was inversely correlated with the copy number of intervals overlapping the region of interest and the aggregate expression of the genes with copy-number loss.

The mutational metric analysis indicated that the micro-deleted astrocytoma subset had significantly less chromosomal instability (as indicated by significantly lower SNV burden, significantly lower CNA frequency, significantly lower degree of aneuploidy and significantly lower frequency of chromothripsis). These observations may indicate that not only the type of oncogenic alteration (causing MYC overexpression) but also the causative mechanism responsible for that alteration type may be different in the 19q13.43 micro-deleted cases.

Deletion of 19q is a common event both in IDH-mutant astrocytomas and IDH-mutant and 1p/19q co-deleted oligodendrogliomas. Such IDH-mutant astrocytomas with 19q loss were associated with better prognosis compared to those without [35]. IDH-mutant astrocytomas with 19q13 have also been reported before, but the authors have suggested that the underlying cause is the loss of the CIC gene, which resulted in oligodendroglioma like appearance [36].

None of these 15 genes are direct interaction partners of MYC as reported by STRING. No direct interactions or relationships were reported in previous literature either. In this study we have not provided a mechanistic analysis of the effect (correlation of 19q13.43 microdeletion with relative MYC overexpression), nor we have confirmed the finding in an independent cohort. Further studies are needed to confirm and uncover the mechanism of this novel observation.

### Supplementary Information

Additional file 1.
