# Supplementary material for: A microdeletion event at 19q13.43 in IDH-mutant astrocytomas is strongly correlated with MYC overexpression
Source: Acta Neuropathol Commun. 2024 Jun 14;12:95. doi: 10.1186/s40478-024-01811-1 (PMC11177509; doi:10.1186/s40478-024-01811-1)
Supplement: Supplementary file 1 — Additional file 1. [file 40478_2024_1811_MOESM1_ESM.pdf]

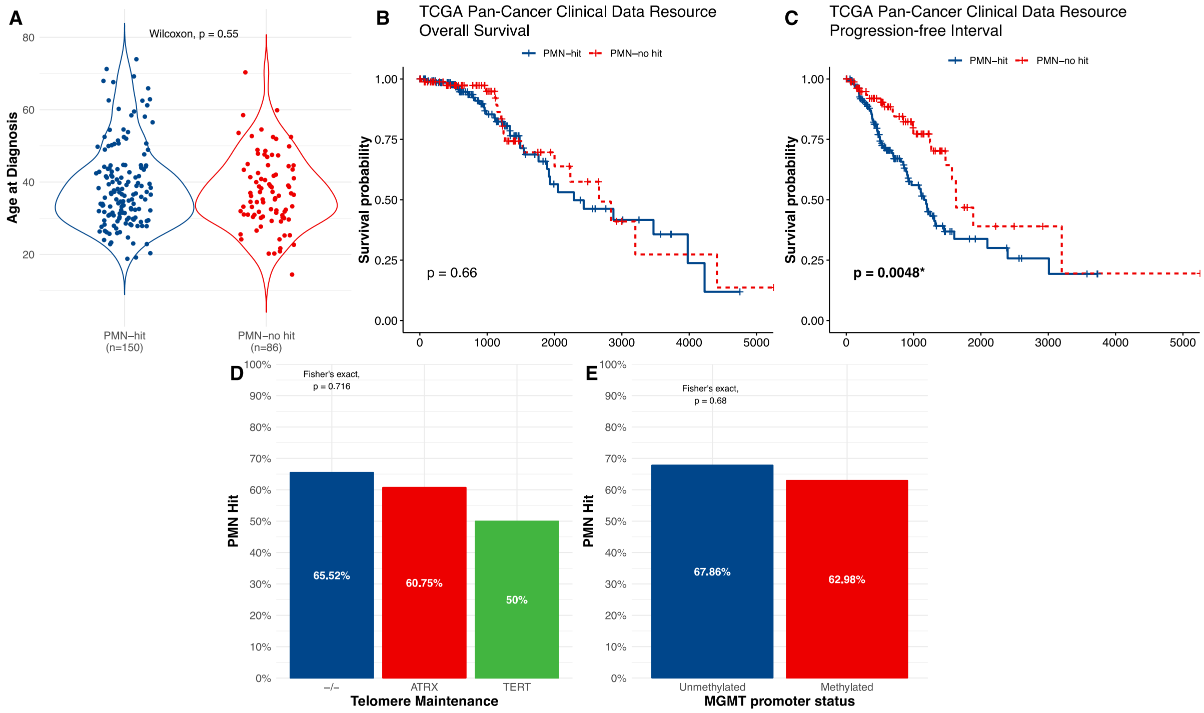

Supplementary Fig. 1: PMN-hit associations with (A) age at diagnosis, (B) overall survival, (C) progression-free interval, (D) telomere maintenance mechanism of the tumour and (E) MGMT promoter status.

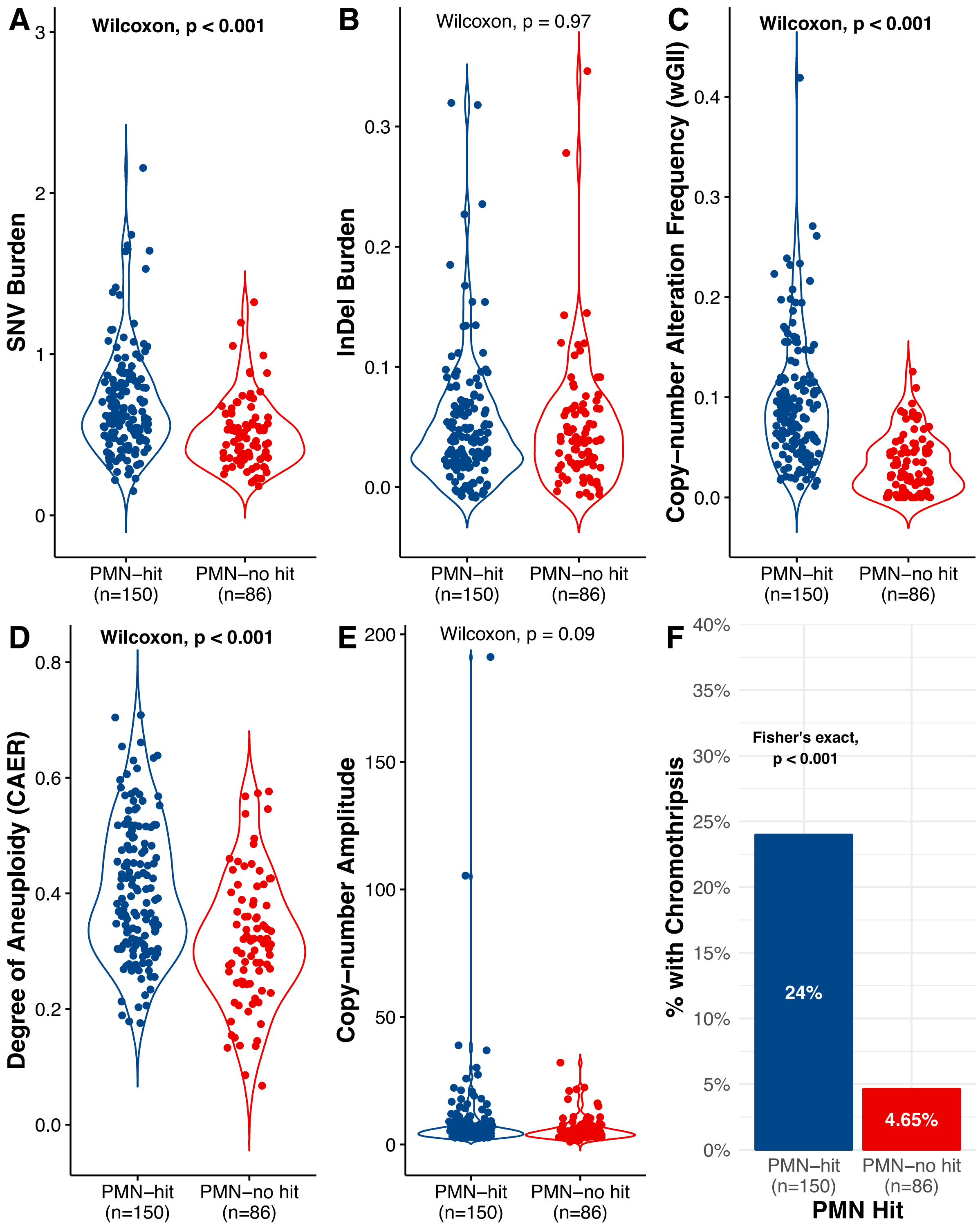

Supplementary Fig. 2: PMN-hit associations with the genomic instability metrics, including (A) SNV burden, (B) InDel Burden, (C) copy-number alteration frequency or weighted genomic instability index (wGII), (D) degree of aneuploidy or chromosomal arm event ratio (CAER), (E) copy-number amplitude and (F) chromothripsis status.

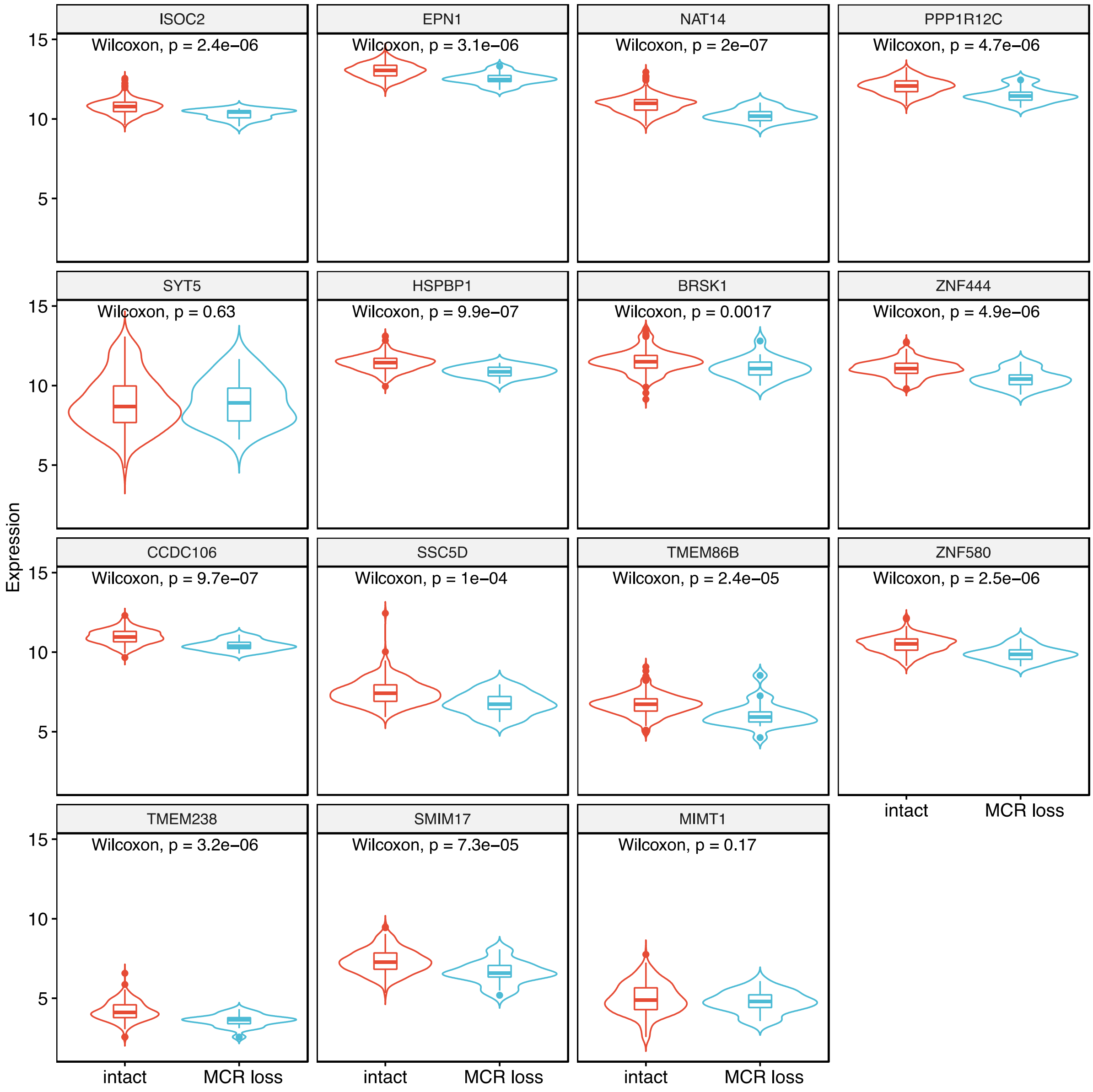

Supplementary Fig. 3: Expression levels of genes (which are associated with increased MYC expression in PMN-WT cases) by MCR loss status (intact vs. MCR loss).

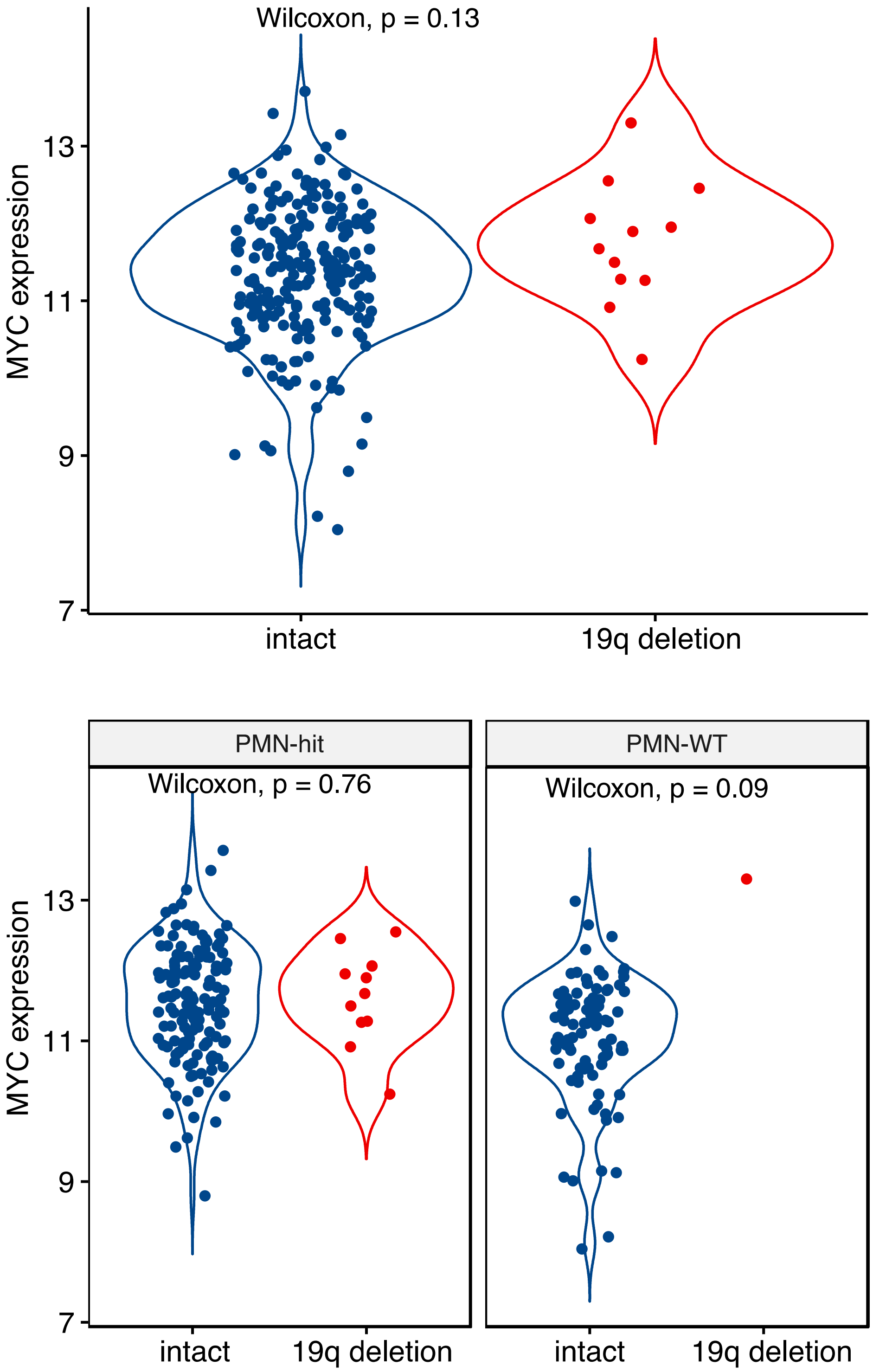

Supplementary Fig. 4: MYC expression and chr19q deletion relationship. MYC expression distribution by chr19q deletion status (19q deletion vs. intact) in (A) all samples and (B) PMN-hit and PMN-WT subgroups.

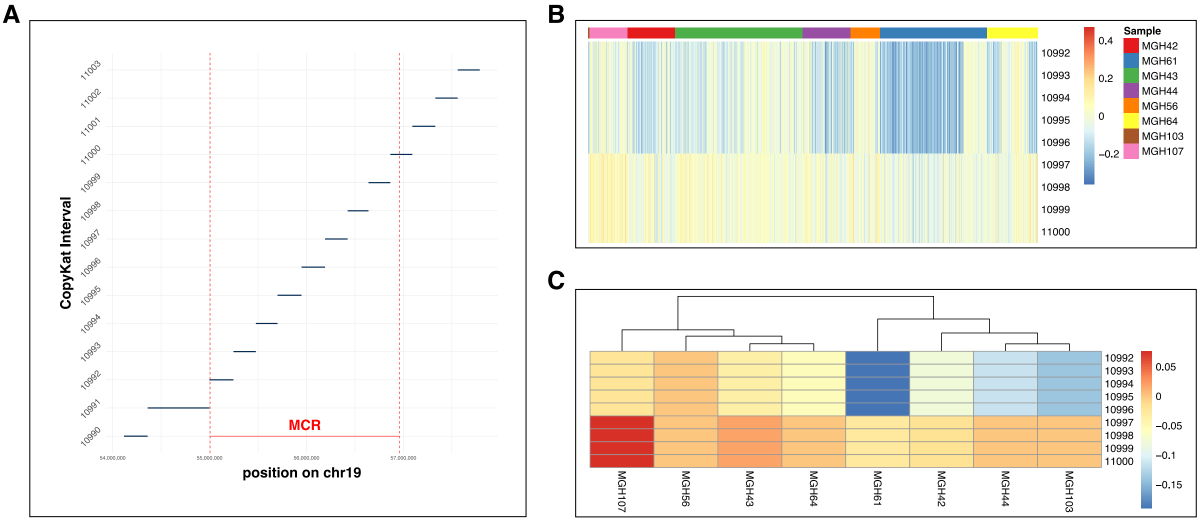

Supplementary Fig. 5: Copy number estimates within the CopyKat windows in the single-cell RNAseq experiment samples (A) The chromosomal positions of the CopyKat windows with respect to the MCR. (B) Copy number estimates (log-ratio values) of windows overlapping the MCR (rows) for each cell per sample (columns). (C) Aggregate copy number estimates of windows overlapping the MCR (rows) for each sample (columns).

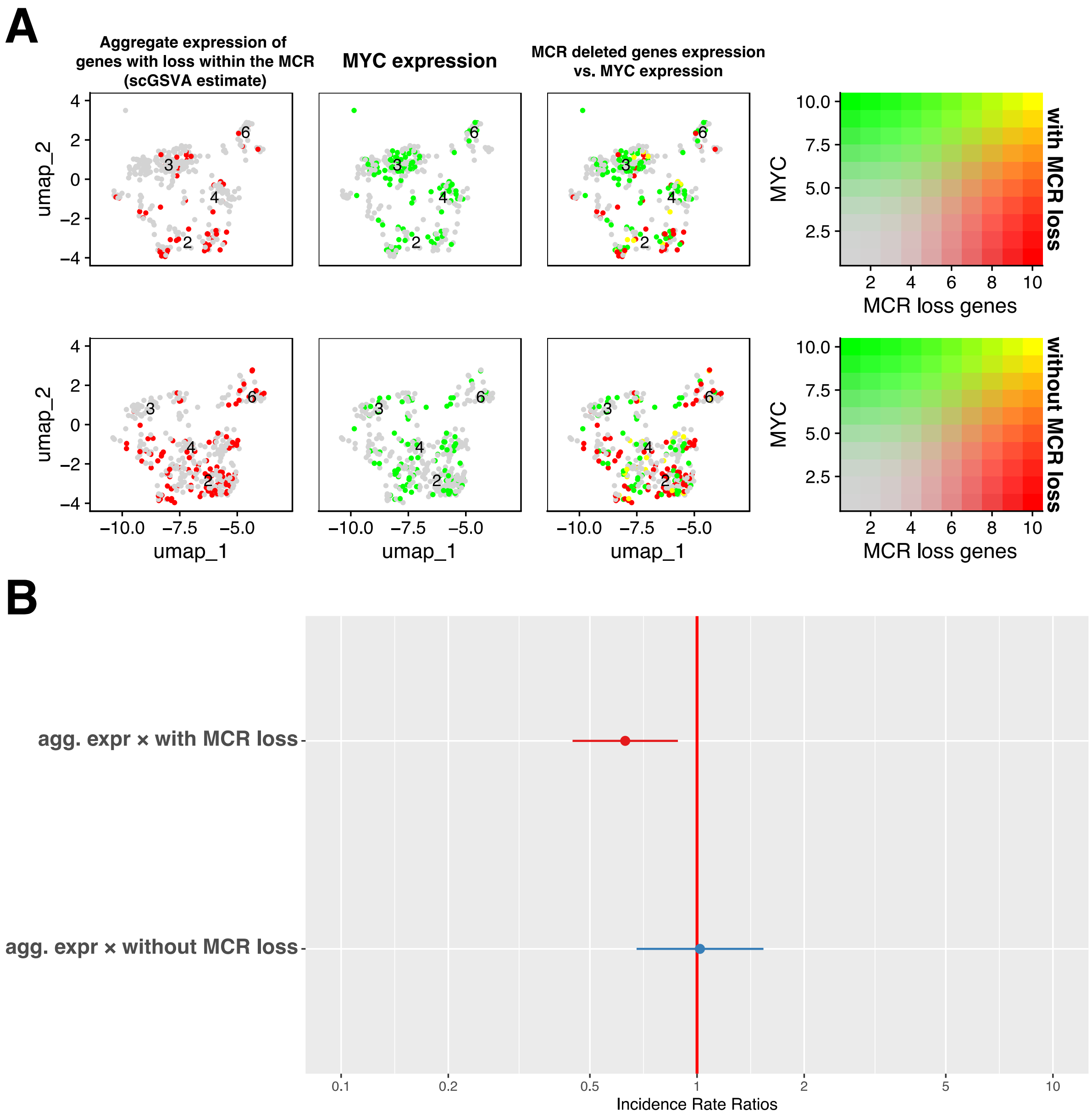

Supplementary Fig. 6: The distribution of the expression of the genes in MCR (aggregated expression) and MYC over the cancer cell population. (A) UMAP plots displaying blended expression of MYC and MCR loss genes (B) coefficients of the Poisson regression model to estimate the effect of aggregated expression of MCR genes (in interaction with MCR loss status) on MYC expression (counts).

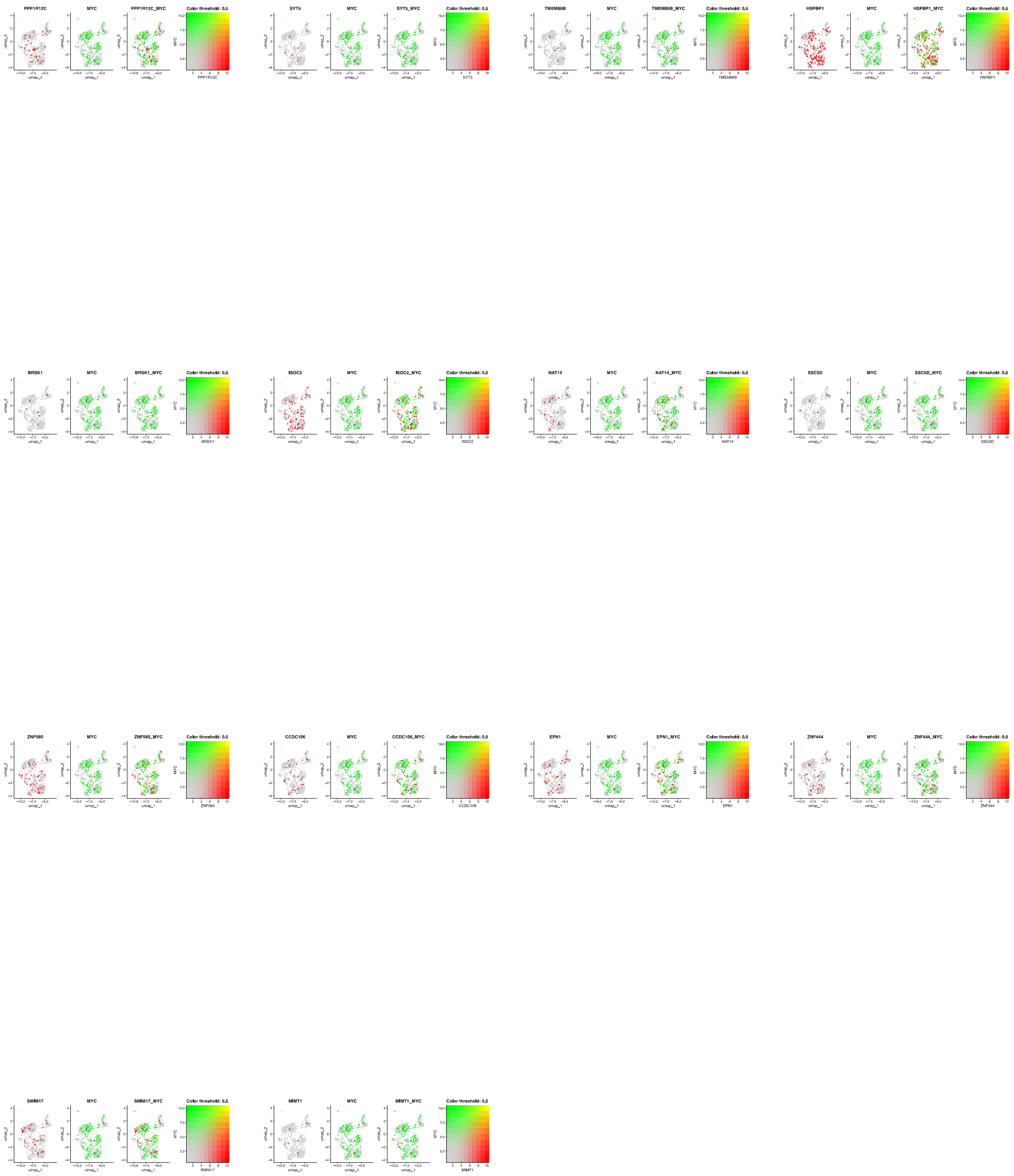

Supplementary Fig. 7: The distribution of the expression of the genes in MCR (each gene individually) and *MYC* over the cancer cell population.
